# Supplementary figures and images for: A Type II hybrid effectiveness-implementation study of an integrated CHW intervention to address maternal healthcare in rural Nepal
Source: PLOS Glob Public Health. 2023 Jan 24;3(1):e0001512. doi: 10.1371/journal.pgph.0001512 (PMC10021605; doi:10.1371/journal.pgph.0001512)

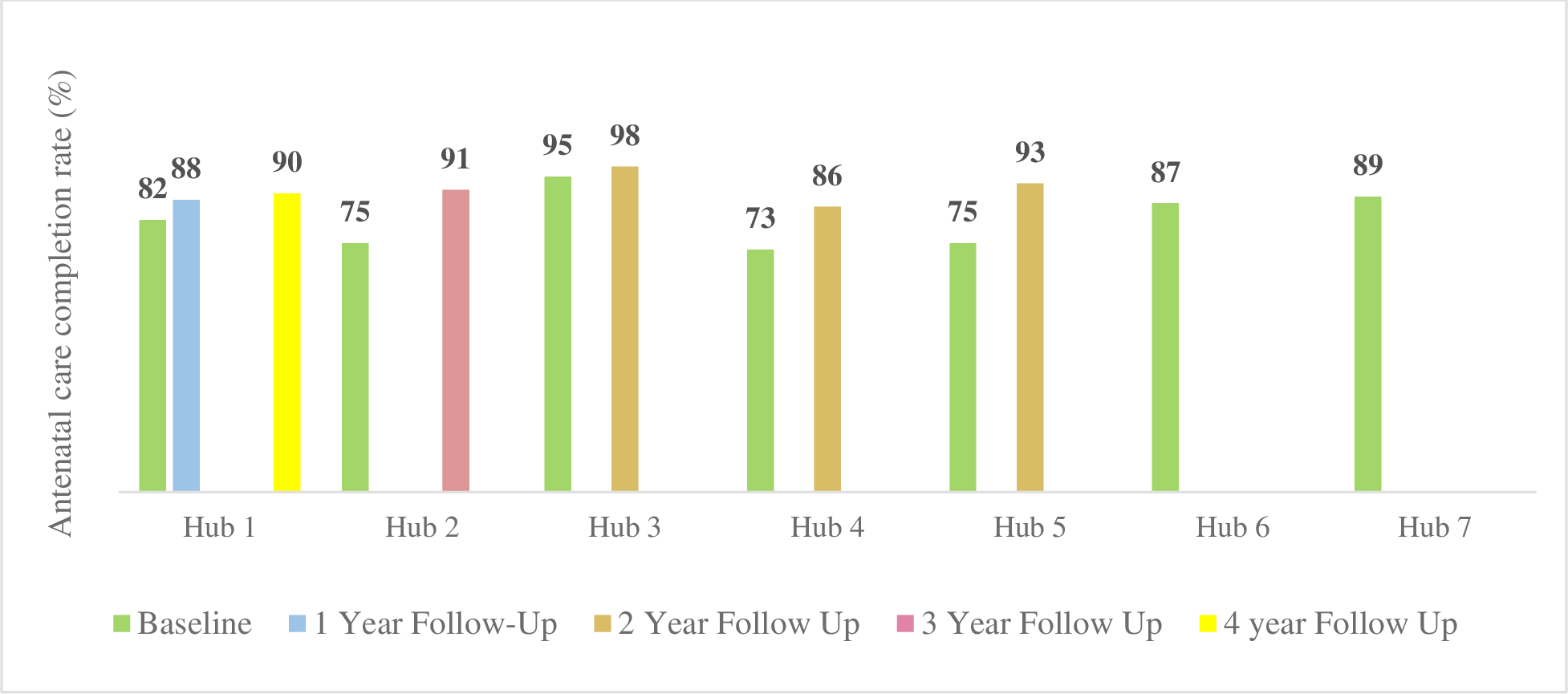

Supplement: S1 Fig — (TIF) [file pgph.0001512.s001.tif]
