# Supplementary material for: A Type II hybrid effectiveness-implementation study of an integrated CHW intervention to address maternal healthcare in rural Nepal
Source: PLOS Glob Public Health. 2023 Jan 24;3(1):e0001512. doi: 10.1371/journal.pgph.0001512 (PMC10021605; doi:10.1371/journal.pgph.0001512)
Supplement: Attachment — Submitted filename: Final Reviewers comments.docx [file pgph.0001512.s003.docx]

**Comments to the Author**

1. Does this manuscript meet PLOS Global Public Health’s [publication criteria](https://journals.plos.org/globalpublichealth/s/criteria-for-publication)? Is the manuscript technically sound, and do the data support the conclusions? The manuscript must describe methodologically and ethically rigorous research with conclusions that are appropriately drawn based on the data presented.

Reviewer #1: Partly

Reviewer #2: Yes

2. Has the statistical analysis been performed appropriately and rigorously?

Reviewer #1: Yes

Reviewer #2: Yes

3. Have the authors made all data underlying the findings in their manuscript fully available (please refer to the Data Availability Statement at the start of the manuscript PDF file)?

The [PLOS Data policy](https://journals.plos.org/globalpublichealth/s/data-availability) requires authors to make all data underlying the findings described in their manuscript fully available without restriction, with rare exception. The data should be provided as part of the manuscript or its supporting information, or deposited to a public repository. For example, in addition to summary statistics, the data points behind means, medians and variance measures should be available. If there are restrictions on publicly sharing data—e.g. participant privacy or use of data from a third party—those must be specified.

Reviewer #1: No

Reviewer #2: No

4. Is the manuscript presented in an intelligible fashion and written in standard English?

PLOS Global Public Health does not copyedit accepted manuscripts, so the language in submitted articles must be clear, correct, and unambiguous. Any typographical or grammatical errors should be corrected at revision, so please note any specific errors here.

Reviewer #1: Yes

Reviewer #2: Yes

5. **Review Comments to the Author**

Please use the space provided to explain your answers to the questions above. You may also include additional comments for the author, including concerns about dual publication, research ethics, or publication ethics. (Please upload your review as an attachment if it exceeds 20,000 characters)

Reviewer #1: This study assesses the effectiveness and the implementation of an evidence-based integrated maternal and child health intervention delivered by CHWs, using a mixed method. Data and statistical methods used are not clearly described. The document does not present clearly the characteristics of participants, which could also be associated with the outcome variables. It is important to consider seasonality of data collected because fertility and child mortality could vary by month.

**Thank you for your suggestion. We described our data and statistical methods in the “Data Collection” and “Data Analysis” sections of the manuscript. We added to each of these sections to make the descriptions more clear. Our analysis was at the ward level (Institutional Birth Rate for women clustered within wards, a geographical unit) as opposed to the individual-level Institutional Birth. Therefore, we think the ward-level characteristics presented in Table 1 would be more relevant (characteristics of the families, and services available by ward). As such, we did not look at the individual-level characteristics of each participant. We have included a sentence in lines 238 – 240 to clarify this. As our unadjusted analysis was at the ward-level instead of individual-level, we also adjusted for the confounding characteristics at the ward-level in the Poisson regression.**

**Thank you for your comment on seasonality. Our main outcome in this paper was the IBR as opposed to fertility and child mortality rates; therefore, we did not specifically look into seasons' effects in this study. As we were assessing IBR on a yearly basis, seasonal effects should not account for differences we observed over the course of the study.**

Reviewer #2: Overall, this is an interesting paper that shows excellent use of implementation science in program implementation. I have few comments:

Abstract

Sentence on what it means for wider public health or contribution to general body of knowledge seems to be missing.

**We have added the following sentence to the end of the abstract: “This study contributes to the growing body of evidence on the role of community health workers in achieving universal health coverage.”**

Introduction

Line 108 - What ‘contextual challenge’ is being referred to please be more specific.

**Thank you for your suggestion. We have specified relevant contextual challenges to our setting from the referenced paper, as suggested.**

Line 112/113 – please add citation.

**We have added the citation, as suggested.**

Line 131 – what were the retention challenges? Also add citation.

**Thank you for bringing this up. The statement was anecdotal since the program and study findings have not been made publicly available yet, as far as we know. Since it was anecdotal, we removed this sentence and noted the impact of the program was unclear.**

Line 135 – Did the project look at specific components of evidence-based intervention? If so, please add.

**In this line, we were referring to the generalized WHO recommendations as opposed to any-specific evidence-based interventions. Our intervention integrated several evidence-based interventions, and in this analysis, we looked at the effectiveness and implementation of the whole integrated package rather than individual components separately.**

Line 141 – reach number seems to be high. Is this for maternal health? Based on Census and HMIS data, I don’t think total size of two districts will add up to 300,00. Please explain, if there is double counting and what events are counted.

**Thank you for your comment. The initial target population of 300,000 we aimed for was the catchment area population, not the women of reproductive age only. According to the 2011 census data, the total population of Dolakha and Achham were 186,557 and 257,477, respectively [1]. These numbers add up to more than 300,000, but we set this target because the program did not cover all the areas of Achham and Dolakha districts, since the program adoption was the local government's decision, as described in the text.**

Study Population

Line 177 – What was the percentage of excluded population

**As mentioned in the text, we used pregnancy history forms to approximate the number of individuals included. Based on this data, about 2.8% of initially enrolled women were excluded from our analysis either due to no consent or due to age outside the eligible range of 15-49.**

Line 179 - How were the three groups recruited for the study. Please add sampling process. Also, who were included in the program staff, is it just CHPA?

**Thank you for this suggestion. The geographic areas to conduct the focus group discussions were purposively selected based on duration of program implementation in that area, and all CHWs working in that area were invited to the FGD. We added this information on Page 13. The program staff included CHPAs, a program coordinator, program managers, program directors, and program monitoring and evaluation officers. This information is included on lines 296-297.**

Intervention
Line 204 - it the sentence referring to ‘post-partum contraception’, if so specify this.

**Thank you for your careful review. Yes, it was specific to ‘postpartum contraception’ and we have specified this as suggested.**

Line 205 – Please describe what components were included in the mobile tools, was it counselling and referral only or were there other functions as well.

**Thank you for your comment. The mobile tool aided CHWs in counseling, decision support for referrals, and data collection. We had mentioned this in the text in lines 218-219. We have also clarified that all the RMNCH intervention components, including pregnancy screening, ANC, PNC, and early childhood care components were in the mobile tools on Page 10.**

Line 213 – Some of the references in the intervention section seem to be from earlier published articles of the program. Is there a way to clarify this.

**Yes, this is correct. Our team (Maru et al., and Nepal et al.,) published the protocol and costing analysis for this study (which we referenced in this manuscript at multiple places as appropriate). We now are disseminating our outcomes. However, we want to reiterate that we have referenced the previous articles wherever relevant.**

Study design and Implementation approach
Line 230 – Add some description of RE-AIM
It would be good to have table that shows key question for each phase of RE-AIM to help reader understand overall objective. This can include process and target respondent for each phase

**Thank you for your suggestion. We have added a brief description and overall objective of using the RE-AIM framework in lines 241 – 244. Since we have a lot of tables and figures in this manuscript, we decided not to include an additional table for RE-AIM. However, we added descriptions of the specific quantitative RE-AIM indicators used in our study in the ‘data analysis section’ on Pages 14 and 15. For the qualitative portion of RE-AIM, we have added a paragraph about the questions we wanted to understand for each component and their respondents on Page 13 under the ‘data collection’ section. We collected our RE-AIM data mostly during the implementation phase, except for the baseline facility ANC visits data.**

Line 235 – How were the clusters formed? Were there any specific criteria since the number of wards in cluster seems to vary

**'Hubs' or clusters were formed based on the local geopolitical unit, comprised of wards before Nepal's federalization. However, federalization led to the nationwide decentralization and restructuring of local governance. As a result, some wards were redefined or added later in previous geopolitical boundaries where our program was ongoing. To avoid any complexities for the study purpose, we continued grouping the same clusters based on the old structure. We considered the newly added wards, where the program and data collection started later separately, as separate 'hubs’ or clusters for research purposes. Thus, the numbers in the clusters vary in our study. We hope this sufficiently clarifies your question.**

Line 243 – was the enrollment period in each hub continuous through the life of project.

**Thank you for your comment. While CHWs registered and enrolled most of the houses, families, and reproductive-aged women during the initial enrollment period at the program outset, they continued enrolling any other newly identified eligible reproductive-aged women after the initial enrollment period (or during the intervention period). Since CHWs only enrolled houses, families, and eligible reproductive-age women and collected baseline data at the program outset without delivering any intervention, we called the initial period at the program outset “the enrollment period.” Thus, the terminology could have caused confusion, but we would like to reiterate that the enrollment continued after the enrollment period for any new individuals identified.**

Line 234 seem to imply it was not. If so, how were the new pregnancies beyond the enrollment period in a hub managed? What about post PNC period, how were they managed? Were there any follow-up post that period?

**Thank you for bringing this up. As mentioned in the above question, CHWs continued enrolling any other newly identified eligible reproductive-aged women even after the initial enrollment period was complete. If women were identified and enrolled after the intervention had started, they would receive the same ANC and PNC interventions as women enrolled in the ‘enrollment period’.**

Line 248 – what was the overall attrition rate, did it impact the study in any way?

**Since we did our analysis at the ward-level, it did not involve following up with the same women in the pre-and post-intervention periods. Therefore, we did not specifically look at each individual loss-to-follow-up and attrition rate. We noticed a few women permanently migrated to other places and stopped receiving the intervention during the study period in each hub. However, some newer individuals also got enrolled in the study when they became eligible. Therefore, we do not think it should cause significant impacts in our population ward-level analysis.**

Data Analysis
Line 313 – Besides self-reporting, were there any CHWs record/reports that were used throughout the process?

**Thank you for this question. CHWs only collected self-reported information from participants. To try and verify the information, CHWs checked ANC visit cards that participants carried from the facility detailing information about their facility visits. These cards have limited information about prior pregnancies and not all participants would be able to provide their ANC visit card at the time of the CHW visit.**

Study Enrollment
Enrollment of Dalit/Janajati/others in Hub 2 and 7 seems to be higher compared to other hubs. Is there a reason for this?

**Thank you for this question. This is because there are higher proportions of Dalit/Janjati/others people residing in these areas than people of ‘high’ castes.**

Line 357/358 – What was the reason for not being able to process data from three hubs?

**Thank you for your comment. In the first three hubs where implementation occurred, there was a delay in starting to analyze individual enrollment data. As such, new individuals may have been enrolled after the initial “enrollment” period. For the 3 hubs that we began implementing in at a later time, we were able to start individual enrollment data analysis immediately after the enrollment period, and thus we were able to verify the enrollment specifically during this period. We hope this sufficiently clarifies your question.**

Implementation Outcome
Line 397 – It seems women were counted more than once. Please explain.

**The population that we mentioned in line 397 was the overall catchment area population, not just the women of reproductive age. As mentioned above, according to the census 2011 data, the total population of Dolakha was 186,557, and the population of Achham was 257,477 [1].**

Line 463 – What percentage were counted separately? Did this have any impact on the study.

**Thank you for this comment. As you correctly noted, in this indicator, some women who reported high-risk symptoms multiple times in the same pregnancy or reported high-risk symptoms in multiple pregnancies could have been counted more than once. By definition, this implementation indicator was the number of ‘high-risk counseling sessions’ or ‘referrals CHWs made’ each year as opposed to the number of ‘women with high-risk referrals.’ We don’t think this should have any effects on the study. For the effectiveness analysis, we did a ward-level analysis as opposed to an individual-level analysis, and our study did not involve following same women pre- and post-intervention.**

Line 480 – ‘lack of trust’ please mention this in enrollment or in limitation
**Thank you for this suggestion. We have included a sentence in the limitation in lines 644 – 646.**

Mechanism of impact of the intervention
Were there any differences in findings by type of respondents – if so it would be good to specify this.

**Thank you for bringing up this point. We did not note differences in qualitative findings by type of respondents. As mentioned in the limitations in the text, due to COVID-19 restrictions, it was challenging to collect qualitative data from as many participants as we had wanted. So, it was not feasible to compare differences in responses by respondent type.**

Discussion
Line 545 – ‘high reach’ – Was this not by design, earlier table seem to suggest this.

**Thank you for this comment. In line 545, we were referring to the people of underprivileged caste. We could say it was due to design because we wanted to have this program reach everyone, including the marginalized population. However, as mentioned in the text, some similar studies have shown disparities in the utilization of CHW services by marginalized populations even though by design, CHW services were supposed to reach everyone in communities. We wanted to look at whether this was the case in our study setting. Our evidence showed there were no disparities in service utilization by marginalized populations.**

Line 551 – What was the reason for wide range in week of birth?
**Thank you for this comment. Although we were not able to explore this question through formal qualitative interviews due to restrictions with the pandemic, the program staff mentioned some possible reasons for the wide range across hubs. They talked about the differences in emphasis placed on visiting the postpartum mother within a one-week timeframe, and the differences in the workloads of CHWs at different hubs.**

Discussion section seems to have more details on findings. Some section is missing comparison from other published articles or findings from similar contexts.
Also add what this means for wider public health context. How should this body of knowledge be treated.

**Thank you for your suggestion. We included comparative similar or dissimilar findings from other studies, wherever applicable, on Pages 27 and 28. We have separated a paragraph of comparison of other CHW intervention studies and our CHW intervention study in lines 571-575 to make it more visible.**

**We have added a separate section, ‘Conclusion’, to add the significance of our study findings for wider public health. We mentioned how our findings could be useful for other similar rural settings by providing evidence that CHW systems in line with the WHO recommendations may be effective in reducing maternal health disparities and improving maternal health care to help achieve universal health coverage.**

6. PLOS authors have the option to publish the peer review history of their article ([what does this mean?](https://journals.plos.org/globalpublichealth/s/editorial-and-peer-review-process#loc-peer-review-history)). If published, this will include your full peer review and any attached files.


**Do you want your identity to be public for this peer review?** If you choose “no”, your identity will remain anonymous but your review may still be made public.


For information about this choice, including consent withdrawal, please see our [Privacy Policy](https://www.plos.org/privacy-policy).

Reviewer #1: **Yes:** Jacques Emina Be-Ofuriyua

Reviewer #2: No

[NOTE: If reviewer comments were submitted as an attachment file, they will be attached to this email and accessible via the submission site. Please log into your account, locate the manuscript record, and check for the action link "View Attachments". If this link does not appear, there are no attachment files.]

While revising your submission, please upload your figure files to the Preflight Analysis and Conversion Engine (PACE) digital diagnostic tool, <https://pacev2.apexcovantage.com/>. PACE helps ensure that figures meet PLOS requirements. To use PACE, you must first register as a user. Registration is free. Then, login and navigate to the UPLOAD tab, where you will find detailed instructions on how to use the tool. If you encounter any issues or have any questions when using PACE, please email PLOS at [figures@plos.org](mailto:figures@plos.org). Please note that Supporting Information files do not need this step.

**References**

1.	Central Bureau of Statistics, United Nations Population Fund. Population Monograph of Nepal. Kathmandu, Nepal; 2014.
